# The Experience of Limited Access to Care for Community-Based Patients With Spinal Cord Injury and Stroke in Nepal and the Potential of Telerehabilitation: A Qualitative Study

**DOI:** 10.1177/00469580221146830

**Published:** 2023-02-17

**Authors:** Mandira Baniya, Chanda Rana, Raju Dhakal, Sophie G Makower, Stephen J Halpin, Ram Hariharan, Manoj Sivan, Matthew J Allsop

**Affiliations:** 1 Spinal Injury Rehabilitation Center, Kavre, Nepal; 2 Leeds Community Healthcare NHS Trust, Leeds, UK; 3 Leeds Teaching Hospitals NHS Trust, Leeds, UK; 4 University of Leeds, Leeds, UK; 5 Sheffield Teaching Hospitals NHS Trust, Sheffield, UK

**Keywords:** rehabilitation, Nepal, spinal cord injuries, stroke, telerehabilitation

## Abstract

This study explores the experiences of care received and management of disability for individuals with spinal cord injury and stroke following discharge from a specialty rehabilitation center, alongside perspectives on the potential role of telerehabilitation. We employed qualitative in-depth face-to-face interviews with patients who had accessed and been discharged from a specialist rehabilitation center in Nepal were used. Interviews sought perspectives of adjusting to, living with, and managing disability alongside the potential role of telerehabilitation in the community setting. Inductive thematic analysis was used to derive themes. A total of 17 participants with spinal cord injuries or stroke were interviewed. Four generated themes included: (i) Difficulties accessing support and perceived mismanagement following initial neurological injury; (ii) Realizing the magnitude and impact of an injury in the absence of clear routes to support; (iii) A multi-faceted symptom burden and its impact; and (iv) The nature and types of interaction with health professionals post-discharge and the potential role of telerehabilitation. We detail accounts of suspended periods with minimal or no support provided from healthcare providers for people with spinal cord injury and stroke following initial acute management. Telerehabilitation could be a worthwhile approach to enhance access to rehabilitation in the community setting but must accompany national efforts to enhance the provision of specialist rehabilitation.

**What do we already know about this topic?**
In Nepal, despite rehabilitation having been included in national health policy and planning, currently specialist rehabilitation services for complex needs is not available in any government hospitals and the needs and experiences of people with disabilities are not well documented.
**How does your research contribute to the field?**
This research addresses a gap in the literature by seeking to understand the experiences of care received and management of a disability for community-based individuals with spinal cord injury and stroke, alongside eliciting perspectives to guide the development of a locally-appropriate telerehabilitation approach for Nepal.
**What are your research’s implications toward theory, practice, or policy?**
Multiple, complex, and unmet needs remain for people with spinal cord injuries and stroke when returning to the community setting in Nepal, with telerehabilitation viewed as an acceptable approach to bridging access to specialist rehabilitation providers.

## Introduction

According to the World Health Organization (WHO),^1^ 15% of people are suffering from disabilities worldwide. In Nepal, the national census reported 513 321 people with disabilities (1.9% of the population), among which 36.3% were persons with physical disabilities.^2^ The healthcare system in Nepal has recently highlighted the need for rehabilitation services and has adopted a 10-year action plan to address rehabilitation needs in the country.^3^ The policy includes a strategy to create, within 5 years, adequate disability and rehabilitation human resources to identify and manage disability. Within 10 years, within every region and state, at least 1 fully equipped rehabilitation center and orthotics/prosthetics workshop is planned.^3^ The capacity of rehabilitation professionals in Nepal is currently very limited with professionals based only in private domestic and international non-governmental organizations.^4^

The impact of a physical disability from spinal cord injury (SCI), stroke, and traumatic brain injury often involves disrupted locomotor function (the most commonly occurring disabilities)^5^ that leads to long-term impairment, including multiple complications, high mortality within 1 to 2 years post-discharge and poor community reintegration and participation.^6^ Rehabilitation is an essential and cost-effective component of health services for people with long-term disabilities,^7^ providing a set of interventions designed to optimize functioning and reduce disability in individuals with health conditions, in interaction with their environment.^8^ A lack of specialist rehabilitation can lead to negative outcomes for people with disabilities, including delayed discharge, limited activities, restricted participation, deterioration in health, increased dependency on others, and decreased quality of life.^9^ There is a critical need to explore approaches to increasing access to specialist rehabilitation care across Nepal, to extend efforts to increase outcomes for patients with disabilities and advance the national rehabilitation strategy.^3^

In the context of low-resource settings, access to rehabilitation services is poorly measured but is typically found to be low.^10^ In Nepal, despite rehabilitation having been included in the national health policy and planning, specialist rehabilitation services for complex needs are not currently available in any government hospitals.^11^ Pathways for community-oriented rehabilitation for individuals with a disability have not been developed following discharge from hospital.^4^ Barriers and challenges to accessing healthcare in Nepal include the mountainous terrains of the region and limited transport infrastructure that can make it particularly challenging for both rural and urban dwellers with disabilities. An approach to overcoming geographical and travel barriers to healthcare access in Nepal involves the use of digital technologies. Digital technologies can be used to facilitate health systems strengthening and support moves toward achieving universal health coverage.^12^ In 2019, the WHO^13^ released guidelines on digital health interventions for health system strengthening. Following a critical evaluation of the evidence on emerging digital health interventions for health system improvements, multiple recommendations for interventions were made. This included using telemedicine approaches to enhance access to health services and using it alongside the existing provision of care.^13^ There remain, however, questions regarding its feasibility, efficacy, and cost-effectiveness in low-resource settings.^14,15^ In Nepal, the coverage of mobile telecommunications includes internet access and use by more than 60% of the population,^16^ with previous evidence suggesting telemedicine was effective among patients in a rural Nepalese community.^14^ The role of telemedicine to support rehabilitation care, also referred to as telerehabilitation, has received limited exploration in the context of Nepal with few examples of its use in the context of LMICs (eg, Bhatta et al^15^ and Bhattarai et al^14^]). The lack of an evidence base underpinning telerehabilitation in LMICs necessitates research to understand the needs and preferences of patients to guide its development as part of rehabilitation care.

This research seeks to address gaps in the evidence base underpinning the delivery of rehabilitation care in Nepal. We aimed to understand the experiences of care received and management of a disability for community-based individuals with SCI and stroke. Furthermore, we sought to elicit perspectives to guide the development of locally-appropriate telerehabilitation approaches as a means of overcoming challenges to accessing specialist rehabilitation care in Nepal. Our approach to eliciting the experiences and preferences of patients seeks to ensure the user-centric design of rehabilitation care in Nepal. Patients are the primary users and beneficiaries of rehabilitation services and telerehabilitation approaches, therefore their engagement and involvement could facilitate the creation of services that meet their needs.

## Methods

A thematic inductive approach with in-depth semi-structured interviews was used to explore experience of community-based people in Nepal following discharge from specialist rehabilitation care, alongside eliciting patient perspectives on telerehabilitation and how it might be used as part of service delivery. This study was nested within a parent study to develop and test a telerehabilitation system to support the delivery of specialist rehabilitation care in Nepal. This study was undertaken to understand the experience of patients living in the community setting at the start of the project, alongside developing requirements to guide the design of the telerehabilitation system in the parent study. We report in accordance with the COnsolidated criteria for REporting Qualitative research (COREQ) checklist.^17^

### Setting

The study was conducted at the Spinal Injury Rehabilitation Center (SIRC), a non-profit, non-governmental organization providing specialist multidisciplinary rehabilitation for people with SCI and acquired brain injury, located in the eastern suburb of the capital city of Nepal, Kathmandu. Patients are referred to SIRC from acute hospitals to receive comprehensive rehabilitation, with referrals taken from across the country. The service is led by a rehabilitation medicine physician, with a multidisciplinary team of staff comprising nurses, physiotherapists, occupational therapists, psychological and peer counselors, vocational training support, social services, community-based rehabilitation practitioners, prosthetists, an orthotist and assistive device technician, and extended services (including recreational therapies and speech and language therapy). In Nepal, there are very few hospitals providing specialist rehabilitation services and SIRC is the largest provider with a capacity of 100 beds and the only specialist rehabilitation center in the country.

### Sample and Sampling

Participants were patients who had previously accessed SIRC and had been discharged between 6 and 12 months prior to the study starting in March 2019. Participants were identified through a review of medical records and purposive sampling was adopted to ensure the representation of participants with a sampling frame including gender, location (rural and urban), and the nature of the disability supported by SIRC (ie, spinal cord injury or stroke).

### Data Collection

Participants were contacted by telephone by the clinical team to make them aware of the study. Individuals who agreed to participate scheduled an in-person visit from a member of the SIRC team at their home (MB and CR), with the team member providing an information sheet and explaining the purpose of the study and what involvement would entail. If participants agreed to participate, written consent was sought. During the initial discussion, it was made clear to potential participants (and their families) that there is no obligation or expectation to participate, and if they choose not to participate that it will not effect their ability to access any other rehabilitation services offered at SIRC. Interviews took place typically with the participant alone for those with SCI. Participants with stroke were also interviewed alone unless requesting the participation of a caregiver. Most of the interviews took place in the community at the home of the participant except for 2 participants’ interviews by telephone which were conducted due to the COVID-19 pandemic and associated lockdown restrictions.

The team members conducting interviews were both females, a trained rehabilitation nurse and a physiotherapist, practicing at SIRC although they had not previously met participants recruited to the study. Both had experience participating in research projects, with 1 researcher (MB) undertaking a master’s degree focusing on health research at the time of the study. Both interviewers were mentored and practiced interviewing with a senior applied health researcher in the team (MJA). At the start of each interview, patient demographic data were collected alongside baseline data for the parent study. A topic guide was developed by all authors and pilot testing with 3 patients at SIRC to ensure ease of understanding of items and the flow of questioning. The topic guide included items relating to participant experiences of living with SCI or stroke from initial onset to the time of the interview, the nature and types of interaction with health services, and the acceptability of telerehabilitation. The intended sample size was informed by the concept of data saturation with a purposive sampling approach.^18^ We sought to recruit between 15 and 20 patient participants (a sample size in which data saturation can be achieved within a focused population^19^) whilst exploring data saturation within code meaning.^20^ Members of the research team (MB, CR, and MJA) monitored data following interviews to determine whether new information was continuing to emerge during the interviewing. After each interview, the interviewers (MB and CR) created summaries of the key content explored and discussed with participants, which was discussed with a third researcher (MJA). New information continued to emerge until the 15th interview, and additional interviews were undertaken to augment the diversity of the sample where possible and ensure full coverage of content within the data corpus from interviews. All interviews were audio-recorded and transcribed.

### Data Analysis

Sample characteristics were descriptively analyzed in SPSS (SPSS: Version 16.0. Chicago, IL, USA). Our qualitative approach to inquiry was reflexive thematic analysis where we inductively identified and developed themes from the data.^21^ Three researchers (MB, CR, and MJA) met several times to develop and refine the codebook using NVivo 12 to manage the data. Initial codes were generated through open coding by 2 members of the research team (MB and CR), with key categories developed through axial coding. These were further refined and formed unique themes derived from the data through selective coding. Themes were discussed and refined through regular discussions with research team members (MB, CR, and MJA) using comparative analysis to guide our exploration and understanding of commonality and divergences within the data, both between codes and themes and between groups of respondents (ie, by condition, gender, and location). Themes were then checked for coherence with coded extracts, reviewed, and refined by members of the wider research team (SM and SJH). A thematic map was then developed by the research team (MB, CR, and MJA) to reflect the themes derived from the analysis.

### Ethical Approvals

Ethical approvals were obtained from the Nepal Health Research Council (ref: 1727; 2nd February 2020) and the University of Leeds School of Medicine Research Ethics Committee (ref; MREC 19-031; 10th December 2019).

## Results

Semi-structured interviews were conducted with 17 participants, including both males (n = 14; 82.3%) and females (n = 3; 17.7%) with spinal cord injuries (SCI) (n = 13; 76.5%)and stroke (n = 4; 23.5%). In total, 21 participants were approached with 4 participants declining to participate. Of the participants with SCI (n = 13; 76.5%), 2 had tetraplegia and 11 had paraplegia. The mean age of participants was 42 years old (ranging from 27 to 70 years). On average, interviews lasted 30 min Caregivers were present for all interviews with patients with stroke. Characteristics of participants are outlined in Table 1. Through comparative exploration of interview data throughout the analysis process, alongside determining that no new aspects, dimensions, or nuances of codes were being identified, it was agreed data saturation was reached. Three themes were generated, including: (i) Difficulties accessing support and perceived mismanagement following initial neurological injury; (ii) Realizing the magnitude and impact of an injury in the absence of clear routes to support; (iii) A multi-faceted symptom burden and its impact; and (iv) The nature and types of interaction with health professionals post-discharge and the potential role of telerehabilitation.

**Table 1.** Overview of Participant Characteristics.

Participants (n = 17)	
Mean age (SD)	42.28 (13.98)
Sex (%)	
Male	14 (82.3)
Female	3 (17.7)
Location (%)	
Rural	11 (64.7)
Urban	6 (35.3)
Terrain (%)	
Hilly	8 (47.0)
Terai	9 (53.0)
Nature of disability (%)	
SCI	13 (76.5)
Stroke	4 (23.5)
Marital status (%)	
Married	11 (64.8)
Single	2 (11.7)
Separated	2 (11.7)
Widower	1 (5.9)
Divorced	1 (5.9)
Employment status (%)	
Unemployed	12 (70.6)
Employed	4 (23.5)
Retired	1 (5.9)
Ability to leave house (%)	
Without assistance	7 (38.9)
With assistance	6 (33.3)
No, confined to home	4 (27.8)

### Difficulties Accessing Support and Perceived Mismanagement Following Initial Neurological Injury

The mechanisms of neurological injury in this cohort included traumatic and non-traumatic SCI and stroke. The participants with stroke were older on average and typically had medical comorbidities (eg, hypertension and diabetes). Those with traumatic injuries reported that, following a fall or road traffic incident, they were assisted by passers-by and taken to either a primary health center, health posts, or emergency departments of general hospitals. Participants reported transfer techniques such as being lifted by their hands and legs and taken to an emergency department in a taxi without proper stabilization.

*“The technique used by the policemen to transfer me was not good, they just lifted me and put me in a taxi and did not provide good treatment until I reached the hospital”.* Male, SCI, urban community

Participants with SCI arising without trauma mostly reported experiencing initial symptoms of mild intermittent low back pain which gradually progressed to loss of muscle power in the lower limbs. For these participants, there was no expectation that symptoms could worsen to the extent of paralysis.

*“Had I paid attention and taken serious concern of my initial low back pain, had I looked for early medical intervention, I would not have been paralyzed”.* Male, SCI, rural community

At the district and provincial hospitals, individuals with SCI reported being kept on conservative management (ie, not being offered or accessing surgical interventions) for between 10 and 21 days. For some participants, this led to the need to seek out support at specialized trauma hospitals in Kathmandu where it was possible to access treatment, such as surgery.

*“It [general hospital] was a very expensive hospital, and they took 21 days but didn’t decide whether I need an operation or not. So we decided to go to Kathmandu. The doctors in Kathmandu told us that my case was critical. Finally, I was operated on in Trauma Centre”.* Male, SCI, rural community

There were multiple challenges reported in accessing support from district and provincial hospitals. Some participants opted to change hospitals as they were not allowed to meet their families and were kept in isolation. Even in major trauma hospitals in Kathmandu, participants were referred from 1 hospital to another because of a lack of facilities and equipment (eg, the capability to undertake magnetic resonance imaging). Participants also reported having to wait for long periods to undergo operations; an experience commonly reported for government-funded hospitals. Consequently, some participants reported seeking treatments at private hospitals for early intervention but this came at a great financial cost to them.

*“I was taken to National Trauma Center [a government hospital], but my family transferred me to [private hospital] because we had to wait a long time to get me operated on. But I was not comfortable in that hospital and I was more depressed because they wouldn’t let me meet my family and kept me mostly in isolation. So, my surgery was done in B & B hospital [private hospital]”.* Male, SCI, urban community

For some participants, perceived poor management of their condition was reported. One participant reported sustaining an SCI when he suddenly fell unconscious. At the hospital, they received a CT scan as part of acute treatment to determine the cause of the unconsciousness. The scan appeared normal so there were no subsequent investigations pursued, and no management was put in place for a suspected spinal injury. The participant expressed that the care team were surprised and concerned the next day when he reported he could not move either of his legs and is now a complete paraplegic.

*“The hospital did a CT scan of the brain and other investigations but they forgot to do an x-ray of the spine because I didn’t sustain any visible trauma in my back. The next day when I couldn’t move my legs, they took me for an x-ray and found that I had a fractured spine”.* Male, SCI, rural community.

### Realizing the Magnitude and Impact of an Injury in the Absence of Clear Routes to Support

When returning home following initial management, participants had high expectations of recovery, believing that repeated investigations meant a good prognosis and chance of recovery. Some participants persisted in undergoing numerous operations in the hope of better outcomes. For most participants, no significant change to their condition was realized.

*“The nerve compression was still present in repeat MRI and second surgery, with no relief in pain and the bowel problem remaining. I was so frustrated and told the doctor to please suggest which hospital I should go to spend more money if he cannot treat me. The doctor suggested to me that further surgery would not benefit me rather I should focus on symptomatic management of my problems”.* Male, SCI, rural community

For participants with SCI, following initial management in an acute setting, often they returned home without education on SCI or an understanding of preventative measures for secondary complications. Participants reported a multitude of secondary complications that were experienced following their initial return home, including pressure injuries, repeated urinary tract infections, neuropathic pain, and contractures.

*“I was sent back home without any knowledge of SCI. At home, I was completely bedridden for two years. Though I was paraplegic I was living a life of a high tetraplegic being so dependent on two to three people for my daily activities. I just lay on my bed and counted the days of my survival. I also developed a pressure ulcer as a result”.* Male, SCI, rural community

Some individuals with pressure injuries initially tried to manage their wounds with conservative treatments at home such as lying in a prone position and wound dressing. When wounds did not heal as expected participants again sought treatments across district and provincial hospitals. This often led to incurring financial payments for participants and their families without satisfactory outcomes.

*“When the pressure ulcer didn’t go even after nine months of prone lying, I decided to go to Kathmandu for further management. I was admitted to one of the leading hospitals in Kathmandu and spent around 1000 USD in 10 days, but no improvement in my pressure ulcer”.* Male, SCI, rural community

Participants with SCI and stroke reported that the impact of the disability has a significant impact on their lives and those of their families. Functionally, participants were dependent on family members for most of the activities of daily living. Furthermore, a lack of environments that are accessible for a wheelchair created a barrier to meeting wider family and friends or participating in recreational or religious activities.

### A Multi-Faceted Symptom Burden and Its Impact

Participants reported a range of problems that frequently occurred in the home environment. including neuropathic pain, spasticity, and bowel and bladder problems, Neuropathic pain was one of the most commonly reported problems experienced by people with SCI. It led to disturbed sleep, emotional discomfort, and difficulty in carrying out activities of daily living; these had a negative impact on their daily experience.

*“There is a lot of impact of pain in my daily life, sometimes I can’t even do my own bowel and bladder management due to severe pain and also I cannot sleep because of pain. I need to travel in search of medicine and treatment, spend a lot of money but still the problem is only a little better or the same”.* Male, SCI, rural community

Pain was reported as a consistent and prominent challenge, with participants exploring and seeking guidance on approaches to managing their pain, including exercises, massage, positioning, prescribed medications, and Ayurvedic treatments.

*“I went to various parts of India and Nepal for other kinds of medicine, some Ayurvedic medicine sellers advised me for some kind of oil with herbs and plants cooked together [for massage]. I tried many of those compositions, but instead of helping my burning pain was worse”.* Male, SCI, rural community

Spasticity was also a common problem experienced by participants with SCI and stroke survivors. Excessive spasticity interfered with daily activities including the use of the toilet, transfer, and walking activities. Excessive spasticity problems were also reported by individuals who had bladder issues such as urethral bleeding and malodorous discharge.

*“Spasticity is most troubling for me right now. It affects me in walking and sleeping. I walk around one to two hours daily. While beginning to walk, spasticity decreases a little but it increases again forcing me to stop, but I have not stopped to fight back this spasticity”.* Male, SCI, urban community

Some participants reported that to manage their spasticity, they found that standing, walking, and stretching activities were beneficial.

*“My spasticity decreases and I can do my activities of daily living with ease after I stand and walk for one or two hours per day”.* Male, SCI, urban community

Problems frequently experienced by participants were difficulty managing water intake and fixed catheterization timing, alongside a lack of wheelchair-accessible toilets in public places. Participants who were reliant on family members for assistance with using the toilet reported that this was a burden on their family and a source of frustration for them.

*“I catheterize myself every 3 – 4 hours and sometimes it has very little urine and sometimes it leaks already, I need to be aware all the time, look at the watch constantly and concentrate on my bladder and catheter. I wish I could be carefree as I used to be before. In public places, someone has to help me get out of the toilet as most of the public toilets are not wheelchair friendly. I need to think before doing anything new”.* Male, SCI, urban community

Participants also expressed concern and stress with not being able to access medication for better bowel and pain management and its high cost. The reported reliance on family members, difficulties with self-management, and financial impact may underpin the common desire to achieve independent bowel and bladder management by participants, often prioritized above being able to walk.

*“I have bladder problems, had urethral bleeding twice already and I am on Foley’s catheter now. My current goal is proper bladder management, though I can walk for two hours”.* Male, SCI, urban community

For participants with stroke, communication difficulties were reported that could lead to withdrawal from socializing due to frustration with symptoms arising from their disability.

*“He needs time to understand the question and try to answer, but the listener keeps on changing the topics and adds another question before he has finished answering one. Such a situation brings a lot of irritation in him, so he avoids meeting people and making a conversation”*. Female, caregiver (for a participant with stroke), urban community

The challenging management of symptoms and sequelae from participants’ injuries were reported to have a sustained and continuing negative impact on the lives of some participants, with emotional, psychological, and relationship challenges reported.

*“I had conflicts with my wife, so we are separated. We live in the same house but on different floors. She does not help me with anything. I get help from my father”.* Male, SCI, urban community

The type of psychological response and the severity of response to living with a disability varied from individual to individual. Where it was possible to do, communicating with friends and family, listening to music, playing games on mobile phones, and socializing with friends helped participants to overcome loneliness. A limited number of participants reported personal growth arising from their experience, reporting an acceptance of their condition and its impact.

*“You become disabled only when you cannot plan the future. Now I am completely independent physically, mentally, economically and socially in a wheelchair”. Male, SCI, rural community*

### The Nature and Types of Interaction With Health Professionals Post-Discharge and the Potential Role of Telerehabilitation

Despite experiencing a range of symptoms and issues, most participants reported that options for contacting and accessing rehabilitation services in person were not explored following discharge. For many participants, SIRC was too far away to travel for a visit, and participants were unsure about using mobile phones to contact health professionals based there. Participants would instead visit their closest health center. Some younger participants reported using digital technologies, such as using social media platforms, to target contact with specific rehabilitation professionals. This included contacting nurses or physiotherapists at SIRC for problems such as pain, wound management, and additional exercises.

*“Once I had a reddish area over my sacrum while transferring on the toilet tub. I contacted the nurse at SIRC, and she told me it was a pressure ulcer. So, with their instruction, I laid down in a prone position for 2 days and the redness was gone”. Male, SCI, urban community*

Some participants reported positive experiences where they had been contacted by mobile phone for follow-up by peer mentors from SIRC, who contacted participants to check on their well-being. This acted as a prompt for a small number of participants to re-establish contact with SIRC to obtain basic equipment, such as those related to bowel and bladder care.

*“I got calls from SIRC sometimes and they ask about how I am doing. Not for health problems but I contacted SIRC staff when I could not find some materials such as rubber sheets and asked them where I can buy them”.* Female, SCI, rural community

All participants were receptive to the concept of telerehabilitation. Most participants lived a long distance from the rehabilitation center with the approach potentially facilitating avoidance of long, difficult journeys. Participants reported that buses and taxi drivers are often reluctant to transport wheelchair users, caused by what was perceived as an aversion to the potential risks of transporting a person with a disability. An alternative option is to travel by ambulance, but this mode is very expensive and often cost-prohibitive for participants.

*“This is an extremely important step taken because I feel being a wheelchair user, with poor accessibility, we cannot even access the basic health needs. In this condition, to be able to connect back to the SIRC team will ease so many hassles of transportation and being able to access teleconsultation from home will ease our life to a great extent”.* Male, SCI, urban community

Some participants from rural areas expressed that it would be of benefit to be able to consult staff at SIRC to determine whether a problem being experienced needs immediate management or can be addressed over the coming days. This was seen as a way of decreasing the stress experienced when problems arise.

*“Rather than travelling all the way to SIRC if we can talk on the phone or contact via phone and share our problems. We can get the correct suggestions whether it is a real problem or not to be worried about. We can decide whether to wait for a week or seek immediate admission to a health centre”.* Male, SCI, rural community

Participants shared that even in urban areas, it is difficult to access health services. They considered telerehabilitation as a useful means of accessing health services during emergencies from home. For many, across rural and urban areas, meeting a physiotherapist who would demonstrate new exercise techniques through video conferencing would enhance their ongoing recovery process and potentially assist them in working toward functional independence in aspects of their daily living.

*“I have improved since I am discharged from SIRC. The exercises that I learnt while I was admitted are not enough now. I need the next level of exercise. I stay far away from SIRC and cannot visit to learn new exercises. So, through this telerehabilitation as much as possible I would like to learn about how to make my lower limbs stronger and able to walk”.* Male, SCI, urban community

Additional techniques that may be supported via telerehabilitation included proper dressing techniques or medication for participants with pressure ulcers.

In terms of preferences for how telerehabilitation approaches might be delivered, many participants from urban areas requested the option to schedule an appointment for online meetings to accommodate family members who had jobs. Another approach suggested was that of selecting a fixed topic for discussion on a certain day. In such planned online meetings, participants were keen on the ability to present their problems and those of their caregivers in real-time to the rehabilitation team.

*“I think it would be better to have a specific day, time, and specific topic for discussion as part of the telerehabilitation program that you are going to start. If there is an available team maybe on Saturday or Sunday afternoon for an hour with a pre-informed topic for discussion, we can arrange our time to attend the tele meeting and discuss our problems”.* Caregiver (of participant with stroke), female, urban community

While telerehabilitation was seen as a way that participants may be able to decrease attendance at institutional visits, participants also expressed the value placed on being met face-to-face in the home. This was felt necessary to identify problems that may remain hidden through a mobile phone and enable participants to feel more comfortable sharing their problems.

*“I would also suggest thinking about the possibility to meet the patients in person through home visits, maybe once in 2 to 3 months. There is a different feeling in the patients and the family members like us when we see the doctor or other professionals in a white coat. Patients and caregivers tend to evolve with the problems that they never told each other. There is an environment or the confidence about your problems to be heard and solved as you see the concerned health professional and you want to talk to them about what you are facing or feeling exactly rather than only doing exercises at home by ourselves or talking via phone”.* Caregiver (of participant with stroke), female, urban community

Some participants residing in urban areas expressed a preference for support with procuring relevant online information that may be useful for their condition. According to 1 participant, even though they referred to online websites for information about the management of common problems of stroke, they are not fully reliable. If SIRC were able to identify, curate, and embed information on the identification and management of common problems within telerehabilitation practices, they would feel more confident and trust the resources.

*“If SIRC can develop a mobile application about common problems and therapies related to stroke or spinal injury, we can be updated about various symptoms, how to improve speech, about monitoring the progress and many more things. We use YouTube to learn about such things, but we cannot be assured about the reliability and authenticity of the information from YouTube. In that aspect, whatever information comes from SIRC will be reliable for us”.* Male, SCI, urban community

## Discussion

**Figure 1.**
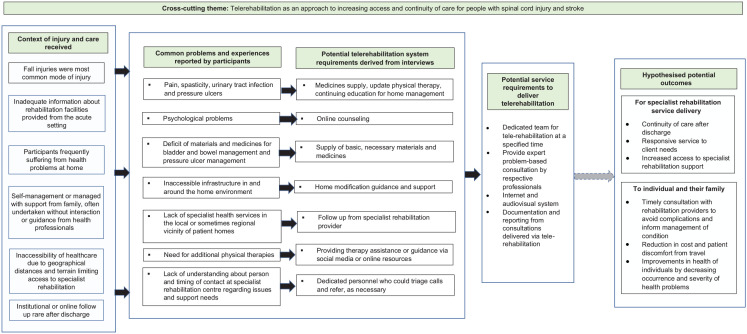
Overview of possible requirements for telerehabilitation to address identified needs of patients derived from study interviews.

Our report provides the first detailing of the experiences of people with traumatic and non-traumatic spinal cord injury (SCI) and stroke and their experiences of accessing care and living in the community in Nepal. Participant accounts detail suspended periods with minimal or no support or clinical input after the initial, acute management of an injury or illness. When living in the community, participants reported exploring self-management of multiple symptoms and concerns with minimal input from health providers. Participants self-managed or were reliant on family, with related physical and psychological sequelae of their injury or illness. This led participants to seek specialist support, enabling orientation, and access to rehabilitation. Despite increased awareness of approaches to the management of their condition, a return to the community was difficult for participants who had limited access to or support from local health facilities. The use of telerehabilitation in the community setting was perceived as an acceptable way of facilitating continued access to continued access to specialist support and expertise at SIRC specialist support and expertise at SIRC as part of post-discharge management, enabling participants to overcome barriers to care including geographical distance and the costs of transport. This work was undertaken as part of a parent study to develop and test a telerehabilitation system to support the delivery of specialist rehabilitation care in Nepal (https://journals.sagepub.com/doi/full/10.1177/11795727221126070). To inform the potential requirements or components that could be explored for telerehabilitation in Nepal in the next phase of the parent project, findings from the study were synthesized into a thematic map (see Figure 1). The figure draws together the identified problems and experiences of participants with possible ways in which telerehabilitation might be used, alongside potential service requirements and hypothesized outcomes from its implementation.

The experience of living with an SCI in the context of a low-income setting includes a high occurrence of pressure sores and urinary tract infections leading to unnecessary suffering, pain, and depression, and often causing premature death.^22^ Stroke, too, is a leading cause of disability and premature mortality.^23^ The current post-discharge care being provided following rehabilitation for SCI and stroke is not addressing the multiple unmet needs reported by participants. Minimal support from local health facilities and the distance and limited options for travel make access to specialist rehabilitation care cost-prohibitive for most people living with physical disabilities. In Nepal, there are currently well-established tertiary care government institutions providing specialist acute care. However, these institutions do not provide specialist rehabilitation. There is a need for the government to consider how to facilitate the expansion of specialist teams providing acute care to specialist rehabilitation services to patients after discharge either face-to-face or virtually, either in their centers or in the community. Globally, rehabilitation and the requisite infrastructure, service development, guidelines, and research have not seen the same levels of progress as acute treatment and management for conditions such as stroke.^24^ Existing institutions investing in specialist rehabilitation could be an interim cost-effective solution in low-resource settings rather than investing in standalone specialist centers for rehabilitation. This model has been shown to be effective in many high-income countries including the UK.^25^ This will require investing in the technology, rehabilitation space, and equipment and staff, alongside the training of individuals and enabling the inclusion of rehabilitation training as part of healthcare professionals’ curricula.

This explorative study suggests that telerehabilitation may provide a useful and worthwhile tool as part of efforts to augment specialist rehabilitation care in Nepal. There is increasing evidence of its potential benefit alongside video-based instruction in the delivery of rehabilitation care in LMICs.^26^ For Nepal, efforts to develop telerehabilitation align with the national e-Health strategy of the Ministry of Health and Population (MoHP) of the Government of Nepal in 2017, with a vision to facilitate equitable and high-quality health care services to enable all Nepali citizens to enjoy productive and quality lives.^27^ Information and Communication Technology as a part of e-Health are identified as having a significant role in promotive, preventive, curative, rehabilitative, and palliative health care services in the context of Nepal. This study articulates requirements derived from patients with SCI and stroke that can be used to guide the design and development of telerehabilitation approaches for the country. As part of the early exploration of telerehabilitation, there will be a need to determine optimal approaches to its role in supporting access to care, including barriers at the patient (eg, cultural beliefs of some regarding the origins and causes of disability, and fear of safety and integrity of accessing and sharing information using digital technologies) and health service (e.g insufficient staff and infrastructure, difficulty navigating the complex application interface) levels which have been identified through telemedicine applications in other areas of healthcare delivery in LMIC settings.^28^ Alongside determining approaches to delivery (eg, frequency, duration, the content of telerehabilitation sessions) there will also be a need to consider sustainability. There is a supportive policy environment for eHealth in Nepal, which could be an important antecedent to the scale-up of telerehabilitation beyond pilot studies. Scale-up of rehabilitation services will require simultaneous development of the provision of rehabilitation care nationally which should include paying specific attention to political (eg, political governance and managerial leadership), professional (eg, increasing knowledge and awareness of the value of rehabilitation), economic (ie, financial support), and sociocultural issues (eg, workforce developments, physical space, and infrastructure).^29^

Our study includes a diverse sample of participants across gender, location, the terrain of residence, nature of their disability, marital status, and employment. This work was completed as part of a larger program of work exploring the design, development, and evaluation of a telerehabilitation system that was deployed in Nepal. However, this research was only conducted in 1 center in Nepal; the only specialist rehabilitation support provided in the country. Our findings therefore may not reflect the experiences of people with spinal cord injuries and stroke who have not accessed rehabilitation services, where there may be needs and experiences differing in their type and magnitude compared to those reported in this study.

## Conclusions

Participants with SCI and stroke in Nepal highlight multiple barriers in accessing and receiving rehabilitation care with a lack of community-based care resources to support the acute and long-term management phases of their condition. A recommendation that can be derived from this study is the need to develop access to specialist rehabilitation support across community settings to respond to the multiple and debilitating needs that are currently unmet for people with SCI and stroke in Nepal. Leveraging digital technologies to develop telerehabilitation approaches was an acceptable approach to participants to bridge access to specialist support, but barriers remain in service capacity and access to technology. Therefore, efforts to develop and implement feasible and clinical- and cost-effective telerehabilitation approaches in the future, may need to be accompanied by support from the Nepalese government to expand the existing provision of specialist rehabilitation care with a particular focus on political, professional, economic, and sociocultural factors.
